# Complete chloroplast genomes of two mint species (Lamiaceae) from Al-Madinah, Saudi Arabia: phylogenetic and genomic comparative analyses

**DOI:** 10.1080/23802359.2022.2130713

**Published:** 2022-10-16

**Authors:** Faten Zubair Filimban, Samaila Samaila Yarádua, Abubakar Bello, Hani Choudhry

**Affiliations:** a Department of Biology, Faculty of Sciences, Division of Botany, King Abdulaziz University, Jeddah, Saudi Arabia; b Department of Biology, Center for Biodiversity and Conservation, Umaru Musa Yarádua University, Katsina, Nigeria; c Department of Science Laboratory Technology, Alqalam University, Katsina, Nigeria; d Biochemistry Department, Faculty of Sciences, Centre of Artificial Intelligence in Precision Medicines, King Abdulaziz University, Jeddah, Saudi Arabia

**Keywords:** Arabian Peninsula, edible plants, genomics, medicinal plants, *Mentha*

## Abstract

The genus *Mentha* encompasses mint species cultivated for their essential oils, which are formulated into a vast array of consumer products. However, the systematics of the genus *Mentha* is very complicated and still uncertain. This is largely because of the presence of frequent interspecific hybridization, variation in chromosome number, cytomixis, polymorphism in morphology and essential oil composition under different environmental conditions, colonial mutant propagation, as well as the occurrence of polyploidy, aneuploidy, and nothomorphs. Here, we present the plastome assemblies for a wilt-resistant Saudi Arabian accession of *Mentha longifolia* (L.) Huds and an alien hybrid *Mentha* × *verticillata* L. which are 152,078 bp and 152,026 bp in length, respectively, and exhibited large single-copy (LSC) and small single-copy (SSC) regions separated by a pair of inverted repeat regions. The chloroplast genome of *M. longifolia* has 133 annotated genes, including 88 protein-coding genes and 37 tRNAs while *M*. × *verticillata* has 133 annotated genes, including 87 protein-coding genes and 38 tRNAs. Both cp genomes have eight rRNA genes. Phylogenetic analysis using a total chloroplast genome DNA sequence of 17 species revealed that *M. longifolia* sequenced in this study did not form a sister relationship with *M. longifolia* from another study. This opens a window for further investigations.

The genus *Mentha* L. (mint) is one of the most significant taxa of the Lamiaceae, and it comprises 24 species, among which 15 are hybrids (POWO). Species in the genus are almost exclusively perennial, herbaceous plants, which are cosmopolitan in distribution. The genus is represented by three species in Saudi Arabia: *Mentha longifolia* (L.) Huds 1762 (locally called Hassawi and Habaq [حبق مديني]), *Mentha pulegium* L. 1753 (Mugrabi) and *M*. × *verticillata* L. 1759 (Al-Madinah mint [نعناع مديني]) with *M. longifolia* being the only known native (Collenette 1985; POWO 2022). All the species are used in herbal teas, alone or as spice mixtures for many foods to offer aroma and flavor. In addition, *Mentha* species had been used for the treatment of many diseases such as throat infection, bronchitis, nausea, and ulcerative colitis (Ahmed et al. 2015; Vining et al. 2017). This makes many *Mentha* species very valuable for industry, as mint oil is a supplement for wide spectrum of products. Other uses of mint essential oils include antipruritic, astringent, antiseptic, and antimicrobial activities, and for treating neuralgia, myalgia, headaches, and migraines (Ahmed et al. 2015; Jedrzejczyk and Rewers 2018).

Despite the significance of the species of the genus *Mentha*, identification and phylogenetic relationships among the species are very complicated and still uncertain. This is largely as a result of the variation in basic chromosome number, frequent interspecific hybridization, cytomixis, polymorphism in morphology and essential oil composition under different environmental conditions, colonial mutant propagation, as well as the occurrence of polyploidy, aneuploidy, and nothomorphs (Gobert et al. 2002; Tucker and Chambers 2002; Denslow and Poindexter 2009; Jabeen et al. 2012; Tucker 2012; Jedrzejczyk and Rewers 2018). This is the cause of the existence of numerous synonymous names (over 3000 published names in the genus), and still uncertain taxonomy of the genus. To solve this problem, there is need to sequence and characterize the plastome of the species.

Two sampled specimens were identified by Dr. Faten Z. Filimban and deposited in the Herbarium of King Abdulaziz University in Saudi Arabia (KAU) (contact info: malgandaby@yahoo.com) including *M. longifolia* (voucher no. FK1021) and *M*. × * verticillata* (voucher no. FK1022) all from Wadi Aqiq near Dhul Hulaifah area, Al-Madinah Province, Saudi Arabia (24.4233720°N, 39.5621330°E and 24.42398°N, 39.56723°E, respectively). The plants were collected in non-protected areas; hence no permission is required for the collection of the species. Total genomic DNA was extracted using plant DNA (BioTeke Corporation, Beijing, China). Genome sequencing was performed on an Illumina HiSeq 2500 platform (Illumina Inc., San Diego, CA). Approximately, 6.0 and 5.1 GB of clean data were obtained for *M. longifolia* and *M.* × *verticillata*, respectively, and assembled using NOVOPlasty (Dierckxsens et al. 2017). The complete chloroplast genome of *Mentha longifolia* (NC_032054.1) was used as seed and reference. The orientation of the assembled sequences was further corrected manually using Geneious prime 2020.0.5 (Biomatters Ltd., Auckland, New Zealand).

The complete cp genome was annotated with the online annotation tool GeSeq and annotations were corrected manually with the sequin v. 15. 50 (Biomatters Ltd., Auckland, New Zealand).

The circular chloroplast genome of *M. longifolia* was 152,078 bp (GenBank accession no. ON124917) in size, contained a large single-copy (LSC) region of 83,220 bp and a small single-copy (SSC) region of 17,652 bp, and separated by a pair of inverted repeats (IRs) regions of 25,603 bp. The cp genome has 133 annotated genes, including 88 protein coding genes, 37 tRNA genes, and eight rRNA genes. The base compositions of the chloroplast genome were uneven (31.5% A, 19.2% C, 18.6% G, and 31.5% T), with an overall GC content of 37.8%.

The complete chloroplast genome of *M. × verticillata* was 152,026 bp in length (GenBank accession no. ON124918) and composed of IRs of 25,605 bp which divide LSC of 83,165 bp and SSC of 17,655 bp, the average GC content was 37.8% (30.7% A, 19.2% C, 18.6% G, and 31.5% T). There are 133 genes annotated, including 87 protein-coding genes, 38 tRNA genes, and eight rRNA genes.

To identify the phylogenetic positions of *M. longifolia* and *M*. × *verticillata* among the species Nepetoideae, we downloaded 13 published complete chloroplast genome sequences of Nepetoideae and chloroplast genome of *Teucrium mascatense* (Ajugoideae), *Congea tomentosa* (Symphorematoideae), and *Dicrastylis parvifolia* (Prostantheroideae) to be used as outgroup to construct the phylogenetic tree. The plastome sequences were aligned with MAFFT v.7 (Katoh and Standley 2013), and analyzed using Bayesian’s inference analysis. The result clearly showed that the tribe is monophyletic based on the samples of species and also supported the division of Salviinae, Nepetinae, and Menthinae subtribes with strong support with posterior probability (PP)=1.0 (Figure 1). The phylogenetic tree revealed that *M*. × *verticillata* is a sister to *Mentha spicata* whereas *M. longifolia* formed a sister relationship with a clade of *M. canadensis* and *M.* × *piperita*. All these relationships were strongly supported (PP = 1.0). Interestingly, *M. longifolia* sequenced in this study did not form a sister relationship with *M. longifolia* from South Africa, this might be as a result of phylogeographic factors. This opens a window for further investigations.

**Figure 1.**
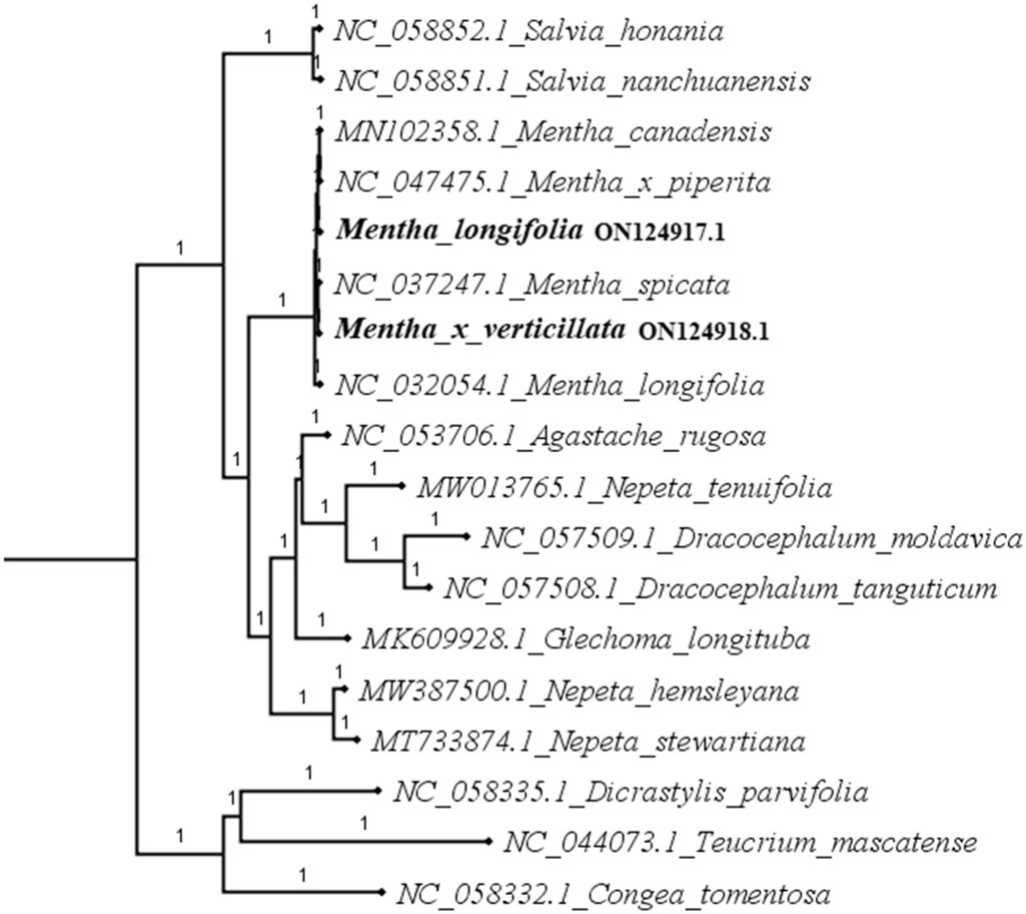
Bayesian’s inference phylogenetic tree of Nepetoideae species (*n* = 13) based on the complete chloroplast genome sequence. Numbers on the nodes represent the posterior probability (PP) values.

## Data Availability

The genome sequence data that support the findings of this study are openly available in GenBank of NCBI at https://www.ncbi.nlm.nih.gov/ under the accession no. ON124917–ON124918. The associated BioProject, SRA, and Bio-Sample numbers are PRJNA842226 for *M. verticillata*, PRJNA842225 for *M. longifolia*, SRR19913734 for *M. verticillata*, SRR20637368 for *M. longifolia* and SAMN28650027 for *M. verticillata*, SAMN28649942 for *M. longifolia*, respectively.
